# Protein Biomarkers in Risk and Prognosis of Amyotrophic Lateral Sclerosis

**DOI:** 10.1111/ene.70741

**Published:** 2026-09-05

**Authors:** Lu Pan, Can Hou, Christina Seitz, Caroline Ingre, Åsa K. Hedman, Jose Laffita, Trung Nghia Vu, Yudi Pawitan, Abbe Ullgren, Solmaz Yazdani, John Andersson, Emily E. Joyce, Charilaos Chourpiliadis, Anikó Lovik, Yan Chen, Sebastian A. Lewandowski, Oscar Fernandez‐Capetillo, Myriam Barz, Kristin Samuelsson, Rayomand Press, Fredrik Piehl, Caroline Graff, Anders Mälarstig, Fang Fang

**Affiliations:** ^1^ Institute of Environmental Medicine, Karolinska Institutet Stockholm Sweden; ^2^ West China Biomedical Big Data Center West China Hospital, Sichuan University Chengdu China; ^3^ Med‐X Center for Informatics Sichuan University Chengdu China; ^4^ Department of Clinical Neuroscience Karolinska Institutet Stockholm Sweden; ^5^ Department of Medical Epidemiology and Biostatistics Karolinska Institutet Stockholm Sweden; ^6^ Pfizer Research and Development Stockholm Sweden; ^7^ Department of Neurobiology, Care Sciences and Society Karolinska Institutet Stockholm Sweden; ^8^ Unit for Hereditary Dementias Karolinska University Hospital Solna Sweden; ^9^ Methodology and Statistics Unit Leiden University Leiden AK the Netherlands; ^10^ Genomic Instability Group Spanish National Cancer Research Centre (CNIO) Madrid Spain; ^11^ Science for Life Laboratory, Division of Genome Biology, Department of Medical Biochemistry and Biophysics Karolinska Institute Stockholm Sweden; ^12^ Networking Research Center on Neurodegenerative Diseases (CIBER‐NED) Instituto de Salud Carlos III Madrid Spain; ^13^ Department of Neurology Karolinska University Hospital Stockholm Sweden

**Keywords:** ALS, diagnosis, Olink, prognosis, proteins

## Abstract

**Background:**

Plasma and cerebrospinal fluid (CSF) protein biomarkers in amyotrophic lateral sclerosis (ALS) may provide insight into disease mechanisms and yield clinically useful biomarkers.

**Methods:**

Overall, 363 proteins in plasma and CSF from 198 patients with ALS and 125 matched controls were profiled using Olink assays. Associations with disease status, survival, and functional decline, as well as longitudinal biomarker stability across the disease course were assessed, together with network and enrichment analyses. ALS risk‐associated biomarkers were externally validated in the UK Biobank (UKB).

**Results:**

Overall, 125 proteins were significantly associated with at least one outcome (i.e., case status, risk, survival, or functional decline), and 21 were associated with three or more outcomes. NEFL was the most robust biomarker in plasma and CSF, alongside TNFRSF12A in plasma and CSF, EDA2R in plasma, and FABP4 in plasma and CSF. Most biomarkers remained stable longitudinally across the disease course. ALS risk‐associated biomarkers were replicated in UKB, in which > 3000 plasma proteins were measured in 52,990 participants, including 298 with ALS. Network and enrichment analyses highlighted their roles in immune response and extracellular‐matrix remodeling, and their enrichments in the brain and T‐cell subsets. Construction of an ALS risk‐prediction model achieved an ROC‐AUC of 0.72 in the UKB validation cohort.

**Conclusions:**

These findings suggest candidate protein biomarkers for ALS risk stratification, early detection, and clinical therapeutic monitoring.

## 1 Introduction

Amyotrophic lateral sclerosis (ALS) is a devastating neurodegenerative disease characterized by the progressive loss of upper and lower motor neurons, leading to muscle weakness, paralysis, and respiratory failure. The global incidence of ALS is approximately 1.59 per 100,000 person‐years, with a prevalence of 4.42 per 100,000 individuals [1]. A higher incidence rate is observed in Europe, ranging from 2.1 to 3.8 per 100,000 person‐years [2]. The average age at diagnosis falls between 60 and 75 years [1], and the male‐to‐female ratio varies across studies and countries [1, 2]. Approximately 10%–15% of patients are familial [3], often linked to pathogenic mutations in genes such as *C9orf72* [4], *SOD1* [5], and *TARDBP* [6] while the majority are sporadic with largely unknown etiology [7, 8]. Most patients die within one to 3 years of diagnosis [9] partly due to diagnostic delay [10, 11, 12]. The limited understanding of the disease mechanisms, clinical heterogeneity, and the absence of curative treatments underscore the need for informative biomarkers to improve early disease detection, risk stratification, and clinical management.

Recent advances in high‐throughput protein‐profiling technologies have enabled systematic characterization of circulating and cerebrospinal fluid (CSF) proteins as potential ALS biomarkers. Previous studies have identified a range of candidate protein biomarkers associated with different aspects of ALS, including disease diagnosis, progression, survival, treatment monitoring, etc. [13, 14, 15]. Among these, neurofilaments, particularly neurofilament light chain (NEFL/NfL) and phosphorylated neurofilament heavy chain (pNfH), are the most established fluid biomarkers in ALS. NEFL are elevated in blood and CSF from patients with ALS and may help discriminate ALS from controls and some mimic disorders, supporting diagnostic utility. NEFL also have strong prognostic value, with strong associations with disease severity and survival [16]. However, neurofilaments are not ALS‐specific, as elevations are also observed in other neurodegenerative or inflammatory disorders [17]. Beyond neurofilaments, several non‐neurofilament protein biomarkers have been proposed, although most remain less validated and their clinical use is less established. Chitinase proteins, including CHIT1 and CHI3L1/YKL‐40, have been reported to be elevated in ALS and have shown both diagnostic and prognostic values, including potential prognostic value when combined with neurofilaments [14, 18]. Another proposed prognostic biomarker is creatinine, which was studied in serum medium [14]. Other candidates include GPNMB [19], complement components [20], GFAP, TDP‐43‐related measures, UCHL1, p75 extracellular domain, and immune‐ or muscle‐related proteins which may have reflected neuroinflammation, complement activation, muscle involvement, or systemic tissue remodeling, but their diagnostic or prognostic utility is generally less established than neurofilaments and often depends on biofluid, assay platform, and clinical context. Nonetheless, many prior high‐throughput protein biomarker studies have been limited by small sample size, analysis of a single biofluid, either plasma or CSF, or cross‐sectional designs with limited longitudinal validation along the disease course [21, 22, 23]. These limitations reduce reproducibility and have hindered translation into clinical practice.

To address these gaps, we conducted a multi‐step Olink Target 96 proximity extension assay‐based protein‐profiling study based on a population‐based case–control study with longitudinal follow‐up, the Swedish ALSrisc Study, and a large prospective cohort for validation and prediction model construction, the UK Biobank. In ALSrisc, plasma and CSF proteins were measured using four targeted Olink panels, Cardiovascular III, Inflammation, Neuro Exploratory, and Neurology. These panels were selected to capture biological processes relevant to ALS pathophysiology, including neuroaxonal injury, neuroinflammation, immune signaling, cardiovascular and metabolic stress, tissue remodeling, and neuromuscular biology. The measured proteins are not ALS‐specific by design but provide targeted coverage of diverse biological domains that may reflect both ALS‐related mechanisms and broader systemic disease‐associated changes. Our objectives were to identify proteins associated with ALS disease status, to assess longitudinal changes during disease progression, and to develop an ALS risk‐prediction model for validation in the UK Biobank.

## 2 Materials and Methods

### 2.1 Study Design

**FIGURE 1:**
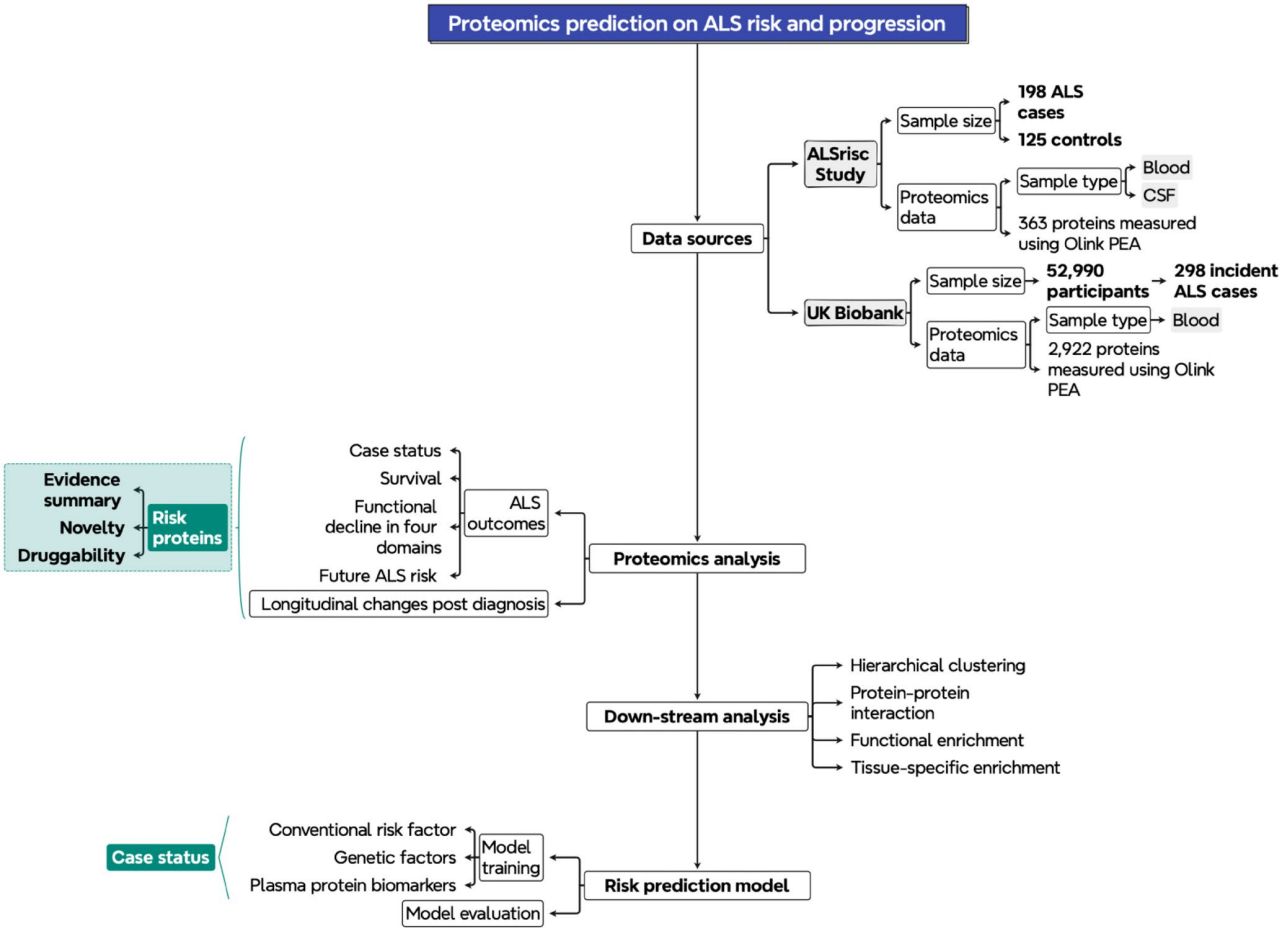
Study design and flowchart.

Figure 1 illustrates the overall study design. We first identify proteins associated with ALS disease status and clinical severity in both plasma and CSF. We further examined longitudinal changes in plasma and CSF protein levels in ALS patients with repeated sampling. We then analyzed the prospective UK Biobank cohort to identify plasma proteins associated with future risk of ALS. Proteins found with implications in ALS were subjected to bioinformatic analyses, including protein–protein interaction (PPI) network construction, functional/pathway enrichment analyses, and tissue‐specific expression profiling. Finally, we developed an ALS risk‐prediction model integrating clinical, lifestyle, genetic, and plasma protein‐profiling features, trained in ALSrisc and validated in UK Biobank.

### 2.2 ALSrisc Study

The ALSrisc (“Biomarkers and Risk Factors for ALS”) Study is an ongoing case–control study based in Stockholm, Sweden [24], enrolling newly diagnosed ALS patients from the Region of Stockholm and their disease‐free full siblings and spouses as controls. Patients are recruited at the time of diagnosis (according to the El Escorial criteria) or shortly thereafter. The present analysis included 198 ALS patients, 78 sibling controls, and 47 spouse controls. The analysis of the ALSrisc Study was approved by the Swedish Ethical Review Authority (DNRs 2014/1815‐31/4, 2018‐1065/31, and 2021‐06397‐02). Oral and written informed consent was obtained from all participants.

Clinical data were obtained through registry linkages and structured interviews. Through linkage with the Swedish Motor Neuron Disease Quality Registry [25], we obtained the diagnosis date, demographics, site of symptom onset, and ALS Functional Rating Scale‐Revised (ALSFRS‐R) scores (recorded at diagnosis and every three months thereafter) of each patient. In addition, lifestyle and medical factors (e.g., BMI, smoking status, history of hypertension, diabetes, or hyperlipidemia) were collected from both patients and controls via a standardized questionnaire modified from the “Euro‐MOTOR” consortium [26].

Plasma and CSF samples were collected from ALS patients at diagnosis and at annual follow‐ups, and from controls at study enrollment (CSF was obtained for a subset of controls). Blood was collected in 10 mL EDTA tubes and centrifuged within 1 h of collection at 2000 g, room temperature to isolate plasma. Genomic DNA was extracted from whole blood using standard protocols. CSF was centrifuged at 300 g, 4°C to remove cells (Data S1). All samples were aliquoted and stored at −80°C until analysis. Samples used in the Olink protein profiling went through two freeze–thaw cycles prior to analysis. The freezing time was calculated as time (days) between sample collection and analysis date (279 days–1682 days). Dedicated assessments of haemolysis and residual platelets were not performed.

Protein profiling in ALSrisc was performed using the Olink proximity extension assay (PEA). In total, 363 proteins were quantified in plasma and CSF using Olink Target 96 Neuro exploratory, 96 Neurology, 96 Cardiovascular III, and 96 Inflammation panels (Table S1) according to the protocols from the manufacturer [27, 28]. Protein abundances were reported as Normalized Protein expression (NPX) on a log2 scale. NPX values were normalized across plates and standardized to z‐scores based on the control group.

Olink NPX values and quality control (QC) metrics were generated according to the workflow of Olink. Assay‐level limits of detection (LOD) are provided in the Data S2, panels A–H. Values below LOD were retained in the main analyses rather than treated as missing, consistent with Olink recommendations, as proteins with low abundance in one group but measurable abundance in another may still provide biologically informative signals. Intra‐ and inter‐assay coefficient of variation provided by Olink were not applied as an additional post hoc protein‐level exclusion criterion. For both plasma and CSF, Olink QC flags were applied at the sample‐protein measurement level. Individual protein measurements flagged as failing QC in specific samples were excluded or treated as missing for those samples. However, no protein assay was removed entirely at the protein level, i.e., all 363 Olink proteins were available across samples and retained for downstream analyses in both plasma and CSF.

ALS patients withdrawn from the study were excluded from the analysis. For samples with technical replicates, replicate NPX values were averaged protein‐wise so that each individual contributed one value per biosample and time point. After sample‐level QC with technical replicates handling, the baseline case–control analyses included 176 ALS plasma samples and 123 control plasma samples, and 165 ALS CSF samples and 14 control CSF samples. Longitudinal measurements up to 36 months were available for 72 ALS patients in plasma and 39 in CSF.

Since *C9orf72* is the most common genetic cause of ALS in ALSrisc, we quantified the length of *C9orf72* G_4_C_2_ hexanucleotide repeat in both ALS patients and controls using a two‐step protocol. Fluorescent PCR was first performed followed by repeat primed PCR [4]. Based on repeat count of the larger allele, the individuals were categorized into four expansion groups: short (≤ 6 repeats), long (7–24 repeats), intermediate (25–144 repeats), and expanded (≥ 145 repeats). Genome‐wide genotyping was performed using the Illumina Global Screening Array, and variants were imputed to the 1000 Genomes Project reference panel. After QC (Data S1), the final genetic dataset included ~7.3 million high‐quality single nucleotide polymorphisms (SNPs) for 170 ALS patients and 120 controls.

### 2.3 UK Biobank Study

UK Biobank is a population‐based cohort of ~500,000 middle‐aged adults recruited across the UK between 2006 and 2010, with baseline phenotyping at assessment centers and ongoing follow‐up through health registries [29]. For the present analysis, we included 52,990 UK Biobank participants with available Olink protein‐profiling plasma data after QC, excluding those with a prior ALS diagnosis. During follow‐up through 2022, 298 of these participants developed ALS. ALS cases were ascertained via hospital or mortality records by ICD‐10 code G12.2 or ICD‐9 code 335.2 for motor neuron disease (MND). Since ALS accounts for approximately 90% of all MND cases [30], MND diagnoses were treated as ALS for this analysis.

Olink Explore 3072 platform was used for the plasma protein profiling by the UK Biobank, covering 2923 unique plasma proteins (Table S2). Details regarding sample handling and QC for the Olink data have been documented previously [31]. NPX values were obtained for 2922 proteins that passed QC (excluding GLIPR1). For this analysis, the NPX values of individual proteins were further standardized to z‐scores based on the mean and standard deviation of the entire cohort.

We obtained the size of the *C9orf72* repeat expansion from the whole‐genome sequencing short tandem repeat (STR) calls that passed QC. Repeat length for the *C9orf72* locus (RapID “C9ORF72”) was extracted for all participants with available Olink protein profiling data, who were categorized into four expansion groups based on the repeat count of the larger allele, as described in the ALSrisc Study above.

Participants were genotyped using the UK Biobank Axiom Array and the imputed genotypes [29] were subjected to standard post‐imputation QC (Data S1), yielding ~9 million high‐quality SNPs for 48,308 individuals.

### 2.4 Statistical and Bioinformatic Analyses

#### 2.4.1 Quality Control of ALSrisc Olink Protein Profiling Data

To assess potential technical artifacts and pre‐analytical variation in the ALSrisc Olink data, we performed additional QC on the z‐score standardized NPX values of plasma and CSF datasets (Table S3). First, we conducted principal component analysis (PCA) separately for each biofluid data and evaluated whether the first two principal components (PC1 and PC2) were associated with ALS disease status or key technical variables, including plate ID and sample storage time. Associations between principal components and covariates were tested using F‐tests from linear regression models. Second, to capture possible pre‐analytical effects, we constructed a pre‐analytical handling variation score based on proteins previously reported to be highly sensitive to such variation [32]. For each sample, NPX values for the available sentinel proteins were aligned and z‐score standardized, then averaged to obtain a single score, with higher values indicating a greater likelihood of pre‐analytical disturbance. We compared this score by ALS disease status and plate ID using analysis of variance (ANOVA).

#### 2.4.2 Analysis of ALSrisc Olink Protein Profiling Data

To identify proteins associated with ALS case status and future ALS risk, we compared baseline protein levels between ALS patients and controls in ALSrisc, and between future ALS cases and non‐cases in UK Biobank. In ALSrisc, generalized estimating equation (GEE) models were applied to account for case–control relatedness, while in UK Biobank, linear regression models were used. All models were adjusted for age, sex, technical factors (plate ID and sample storage time), and other covariates including BMI, smoking, and medical history (hypertension, diabetes, and hyperlipidemia).

We next examined whether baseline protein levels predicted disease progression for ALS patients in ALSrisc. Cox proportional hazards models estimated hazard ratios (HRs) for mortality per standard deviation increase in each protein, adjusting for the covariates stated above with site of onset as an additional covariate. Longitudinal ALSFRS‐R decline was assessed with mixed‐effects models, adjusting for the same covariates.

Among ALS patients with paired longitudinal protein measurements, temporal trends in protein levels up to 3 years after diagnosis were assessed using a linear mixed‐effects model with a random intercept for each patient, adjusting for age, sex, technical factors, BMI, smoking, disease history, and site of onset. *p*‐values were adjusted for multiple testing using the Benjamini‐Hochberg (BH) False‐Discovery Rate (FDR) method [33], with an FDR < 0.05 considered statistically significant.

For proteins significantly associated with ALS outcomes (disease status, risk, survival, and functional decline) (Data S1), PPI network was constructed using Metascape [34], applying the Molecular Complex Detection (MCODE) algorithm to identify tightly connected sub‐networks. Functional enrichment analysis was performed using GSEApy [35] for functional enrichment of the genes of these proteins in canonical pathways [36], transcription factor (TF) target gene sets [37, 38], and disease‐associated gene sets [39]. Additionally, we conducted gene set enrichment analyses (GSEA) [36] to assess tissue and cell‐type specificity using transcriptomic profiles from 46 human tissues [40] and 18 immune cell types [41]. For all enrichment tests, significance was defined as FDR < 0.05. For each identified protein, we quantified prior evidence linking it to ALS and related neurodegenerative conditions using DisGeNET gene‐disease association (GDA) scores [39] and evaluated its druggability by querying the ChEMBL database [42].

#### 2.4.3 ALS Risk Prediction Model for Non‐C9orf72 Carriers

Finally, we developed an ALS risk prediction model for individuals without pathogenic *C9orf72* expansions (< 145 repeats). Predictors included demographic factors (age and sex), lifestyle factors (BMI and smoking), disease history (hypertension, diabetes, and hyperlipidemia), genetic factors (*C9orf72* short, long, and repeat groups, and a polygenic risk score [PRS] for ALS), and plasma protein markers (357 proteins measured in both studies). The PRS was constructed from ALS genome‐wide association study (GWAS) in European ancestry population [43] using SBayesRC [44], excluding the *C9orf72* locus.

NEFL was used as an independent predictor, while the remaining 356 proteins were summarized into composite features. A transformer‐based model TabPFN [45] was used to embed the 356 proteins into a lower‐dimensional space, followed by UMAP [46] to obtain five latent protein embeddings (Data S1). Four nested L2‐regularized logistic regression models were trained on the ALSrisc dataset: basic model comprising age, sex, BMI, smoking, and disease history; basic model with genetic factors; basic model with genetic factors and NEFL; and basic model with genetic risk factors, NEFL, and the five protein‐profile embeddings. Performance was validated in the UK Biobank dataset using area under the receiver operating characteristic curve (ROC‐AUC) and area under the precision‐recall curve (PR‐AUC). SHapley Additive exPlanations (SHAP) values [47] were computed to assess feature importance in the final model.

## 3 Results

### 3.1 Characteristics of the Study Individuals

The ALSrisc Study included 198 ALS patients (50.0% female, mean age at diagnosis 65.5 years) and 125 matched controls (56.8% female, mean recruitment age 62.8 years) (Table 1). Compared to controls, ALS patients had a lower BMI at diagnosis (58.6% vs. 37.6% with BMI < 24.1 kg/m^2^) and were less likely to be never smokers (31.3% vs. 40.0%). Among the ALS patients, 17 (8.6%) were carriers of *C9orf72* pathogenic expansion (≥ 145 repeats). At 3 years of follow‐up from diagnosis, 161 (81.3%) of the patients had died, with a median survival of 14.5 months (Table S4).

**TABLE 1:** Baseline characteristics of participants in the ALSrisc Study and UK Biobank study.

Characteristics	ALSrisc study			UK biobank study	
	ALS cases (*N* = 198)	Spouse controls (*N* = 78)	Sibling controls (*N* = 47)	With ALS diagnosis (*N* = 298)	Without ALS diagnosis (*N* = 52,692)
Age (mean, SD), year	65.46 (11.01)	63.76 (10.12)	61.23 (8.25)	61.05 (6.77)	57.33 (8.21)
Sex, *N* (%)					
Female	99 (50.00%)	46 (58.97%)	25 (53.19%)	135 (45.30%)	28,436 (53.97%)
Male	99 (50.00%)	32 (41.03%)	22 (46.81%)	163 (54.70%)	24,256 (46.03%)
Smoking, *N* (%)					
Never smoker	62 (31.31%)	27 (34.62%)	23 (48.94%)	148 (49.66%)	28,510 (54.11%)
Former smoker	73 (36.87%)	38 (48.72%)	19 (40.43%)	119 (39.93%)	18,361 (34.85%)
Current smoker	51 (25.76%)	8 (10.26%)	1 (2.13%)	Suppressed^a^	5531 (10.50%)
Unknown	12 (6.06%)	5 (6.41%)	4 (8.51%)	< 5^a^	290 (0.55%)
BMI, *N* (%)					
< 24.1 kg/m^2^	116 (58.59%)	26 (33.33%)	21 (44.68%)	65 (21.81%)	12,735 (24.17%)
24.1–29.9 kg/m^2^	69 (34.85%)	45 (57.69%)	21 (44.68%)	154 (51.68%)	26,520 (50.33%)
> 29.9 kg/m^2^	13 (6.57%)	7 (8.97%)	5 (10.64%)	73 (24.50%)	13,189 (25.03%)
Unknown	0 (0.00%)	0 (0.00%)	0 (0.00%)	6 (2.01%)	248 (0.47%)
History of hypertension, *N* (%)^b^					
No	82 (41.41%)	48 (61.54%)	31 (65.96%)	261 (87.58%)	48,060 (91.21%)
Yes	62 (31.31%)	20 (25.64%)	15 (31.91%)	37 (12.42%)	4632 (8.79%)
Unknown	54 (27.27%)	10 (12.82%)	1 (2.13%)	0 (0.00%)	0 (0.00%)
History of hyperlipidemia, *N* (%)^b^					
No	104 (52.53%)	55 (70.51%)	39 (82.98%)	282 (94.63%)	50,637 (96.10%)
Yes	38 (19.19%)	12 (15.38%)	4 (8.51%)	16 (5.37%)	2055 (3.90%)
Unknown	56 (28.28%)	11 (14.10%)	4 (8.51%)	0 (0.00%)	0 (0.00%)
History of diabetes, *N* (%)^b^					
No	136 (68.69%)	62 (79.49%)	39 (82.98%)	290 (97.32%)	51,406 (97.56%)
Yes	11 (5.56%)	6 (7.69%)	7 (14.89%)	8 (2.68%)	1286 (2.44%)
Unknown	51 (25.76%)	10 (12.82%)	1 (2.13%)	0 (0.00%)	0 (0.00%)
*C9orf72* G_4_C_2_ repeat expansion, *N* (%)^c^					
Short (≤ 6)	102 (51.52%)	51 (65.38%)	30 (63.83%)	164 (55.03%)	30,371 (57.64%)
Long (7–24)	51 (25.76%)	23 (29.49%)	14 (29.79%)	97 (32.55%)	21,768 (41.31%)
Intermediate (25–144)	1 (0.51%)	0 (0.00%)	0 (0.00%)	< 5^a^	111 (0.21%)
Expanded (≥ 145)	17 (8.59%)	1 (1.28%)	0 (0.00%)	34 (11.41%)	66 (0.13%)
Unknown	27 (13.64%)	3 (3.85%)	3 (6.38%)	< 5^a^	376 (0.71%)

^a^ For UK Biobank data, cells containing fewer than five participants are reported as < 5 in accordance with UK Biobank disclosure‐control guidance. Additional cells are suppressed where necessary to prevent derivation of small cell counts from reported totals.

^b^ Medical histories of hypertension, hyperlipidemia, and diabetes were ascertained using a standardized questionnaire modified from the Euro‐MOTOR consortium in the ALSrisc Study and using linked health registry data in the UK Biobank.

^c^ C9orf72 G4C2 repeat length was ascertained using fluorescent PCR followed by repeat‐primed PCR in the ALSrisc Study and using whole‐genome sequencing‐derived short tandem repeat calls in the UK Biobank. Expansion categories were defined according to the repeat count of the larger allele.

The UK Biobank study included 52,990 individuals, of whom 298 were diagnosed with ALS during the follow‐up (Table 1). Among ALS cases, 45.3% were female, and median time from baseline to ALS diagnosis was 5.9 years. Thirty‐four cases (11.4%) carried the *C9orf72* pathogenic expansion compared with 0.13% for non‐ALS participants. The ALS cases had slightly higher BMI at baseline compared to other participants (21.8% vs. 24.2% with BMI < 24.1 kg/m^2^).

### 3.2 Quality Control Assessment of the ALSrisc Olink Protein Profiling Data

Key technical and biological characteristics for ALSrisc participants with plasma and CSF proteomic data are, as mentioned above, summarized in (Table S3). PCA of z‐score standardized NPX values showed that plate ID and sample storage time were not associated with the first two principal components in either plasma or CSF, and there was no visual clustering by these technical variables (Figures S1 and S2). In contrast, ALS cases and controls showed modest but statistically significant separation along the first two principal components (Figure S3). The pre‐analytical handling variation scores also did not differ significantly by ALS disease status or plate ID in either biofluid, with similar score distributions across groups (Figures S4 and S5). Taken together, these findings provide no evidence of major technical or pre‐analytical bias in the ALSrisc proteomic data.

### 3.3 Proteins Associated With ALS Case Status and Severity Features in ALSrisc

**FIGURE 2:**
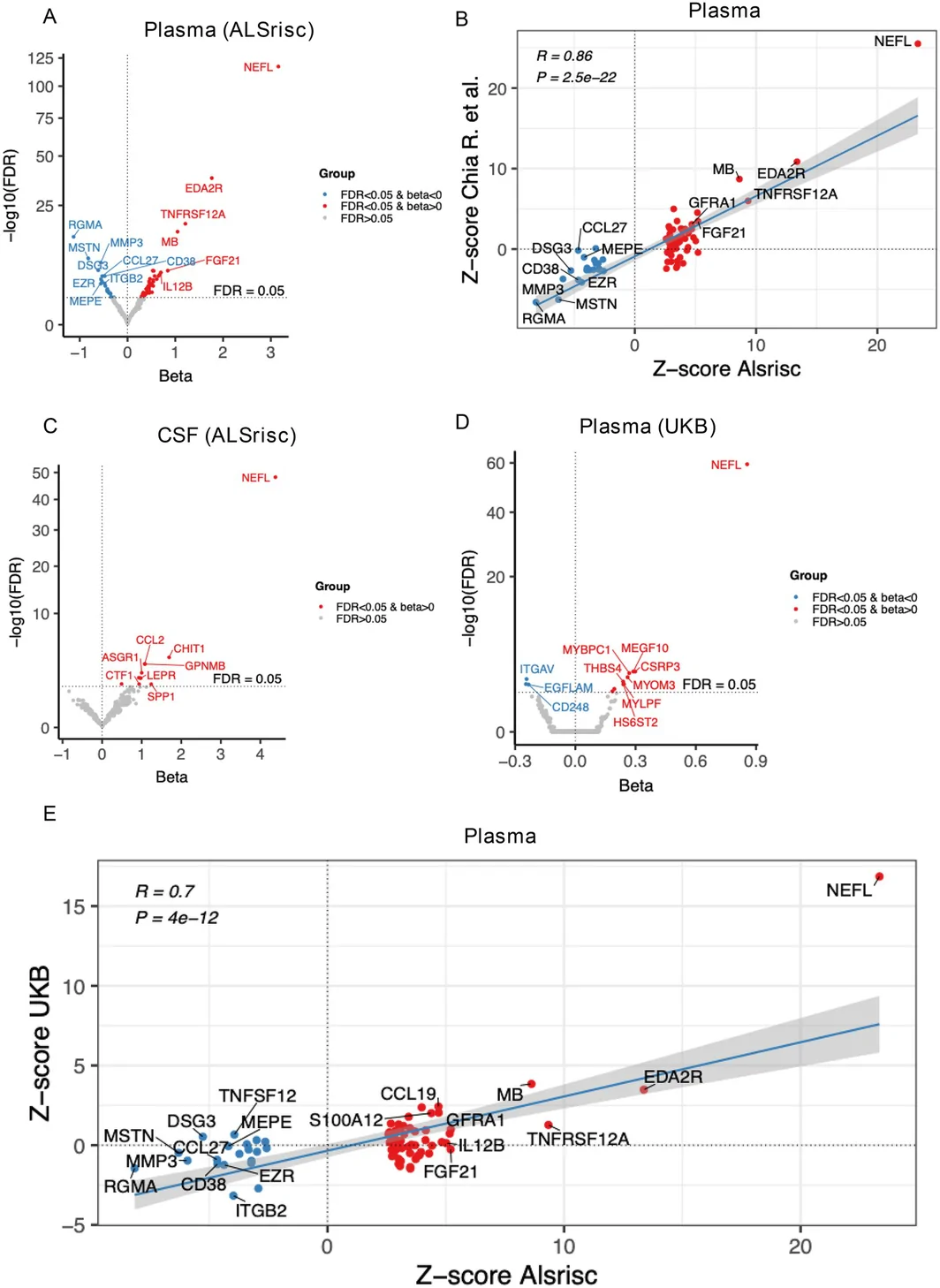
Olink protein signatures of ALS case status and risk of future ALS (A, C): Volcano plots demonstrating the associations of plasma (A) and CSF (C) proteins with ALS case status in the ALSrisc Study. The x‐axis illustrates the adjusted protein level difference between cases and controls derived from generalized estimating equations, and the y‐axis indicates ‐log_10_(FDR). Red dots are proteins significantly up‐regulated in ALS cases (FDR < 0.05, β > 0), blue dots are proteins significantly down‐regulated in ALS cases (FDR < 0.05, β < 0), and gray dots are nonsignificant proteins. Selected top hits are labeled. (B): Scatter plot of z‐scores from the ALSrisc Study (the association analysis of plasma ALS versus control) comparing with corresponding z‐scores reported by Chia et al. [1] The 73 proteins shown were selected from the ALSrisc Study plasma analysis at FDR < 0.05. Fitted linear regression line is shown (blue line) with shading (in gray) indicating 95% confidence interval. Two‐sided Pearson's correlation was used for the comparison. (D): Volcano plots demonstrating the associations of baseline levels of plasma proteins with ALS risk in the UK Biobank study (52,990 participants; 298 incident cases). The x‐axis illustrates the adjusted difference in the baseline protein levels between cases and participants free of ALS derived from linear regression model, and the y‐axis illustrates ‐log_10_(FDR). Red dots are proteins with higher baseline levels in ALS cases (FDR < 0.05, β > 0), blue dots are proteins with lower baseline levels in ALS cases (FDR < 0.05, β < 0), and gray dots are nonsignificant proteins. Selected top hits are labeled (E): Scatter plot of z‐scores from the ALSrisc Study (the association analysis of plasma ALS versus control) comparing with corresponding z‐scores from the UKB cohort. The 74 proteins shown were selected from the ALSrisc Study plasma analysis at FDR < 0.05 which were also been analyzed in the UKB cohort. Fitted linear regression line is shown (blue line) with shading (in gray) indicating 95% confidence interval. Two‐sided Pearson's correlation was used for the comparison.

In ALSrisc, 76 plasma proteins were significantly associated with ALS disease status (FDR < 0.05), of which 54 were upregulated and 22 were downregulated in ALS (Table S5, Figure 2A). Upregulated proteins included NEFL, EDA2R, and TNFRSF12A, while RGMA, MSTN, and DSG3 were among the most downregulated proteins. Exploratory hierarchical clustering was used to visualize co‐expression patterns among ALS‐associated plasma proteins (Figures S6 and S7). Functional annotation of these protein clusters suggested broad involvement of neuroinflammatory processes, tissue injury or repair responses, neuroaxonal injury, muscle‐growth regulation, extracellular matrix organization, TNF‐superfamily signaling, and neural or epithelial adhesion‐related pathways (Figures S6 and S7). Comparison of our ALSrisc panel with that of a recent ALS plasma protein‐profiling study using the Olink Explore 3072 PEA‐based platform [48] identified 73 overlapping proteins, among which 14 were significantly associated with ALS in both studies at FDR < 0.05. Effect estimates across the overlapping proteins showed strong concordance between studies (Pearson ρ = 0.86; Figure 2B). We further evaluated cross‐platform concordance by comparing our results with an independent ALS plasma study profiled using SomaScan [49]. Among ALSrisc plasma proteins significant at FDR < 0.05, five were not measured on the SomaScan panel, and the remaining overlapping proteins showed moderate concordance (Pearson ρ = 0.58), with nine directionally concordant proteins significant in both studies (FDR < 0.05; Figure S8).

In CSF, ten proteins were significantly elevated in ALS patients (FDR < 0.05; Figure 2C, Table S6), of which four also showed concordant elevation in plasma, i.e., SPP1, GPNMB, NEFL, and GDF15. Functional annotation of these concordant proteins suggested involvement of neuroaxonal injury, neuroinflammatory or microglial activation, tissue remodeling, and cellular stress responses, while hierarchical clustering was used as an exploratory visualization (Figure S9).

Similar analysis was performed for UK Biobank data. In total, 13 plasma proteins were significantly associated with future ALS risk (FDR < 0.05; Table S7), including ten Proteins that were elevated whereas three proteins were decreased at baseline among patients who later developed ALS (Figure 2D). Overall, the z‐scores observed for case status in ALSrisc and future ALS risk in the UK Biobank were highly correlated (Pearson ρ = 0.70; Figure 2E). Of the 74 plasma proteins with an FDR < 0.05 found in ALSrisc that had been measured also in the UK Biobank, 47 exhibited the same direction of association in both studies, whereas nine proteins were nominally significant (*p* < 0.05) in the UK Biobank study.

**FIGURE 3:**
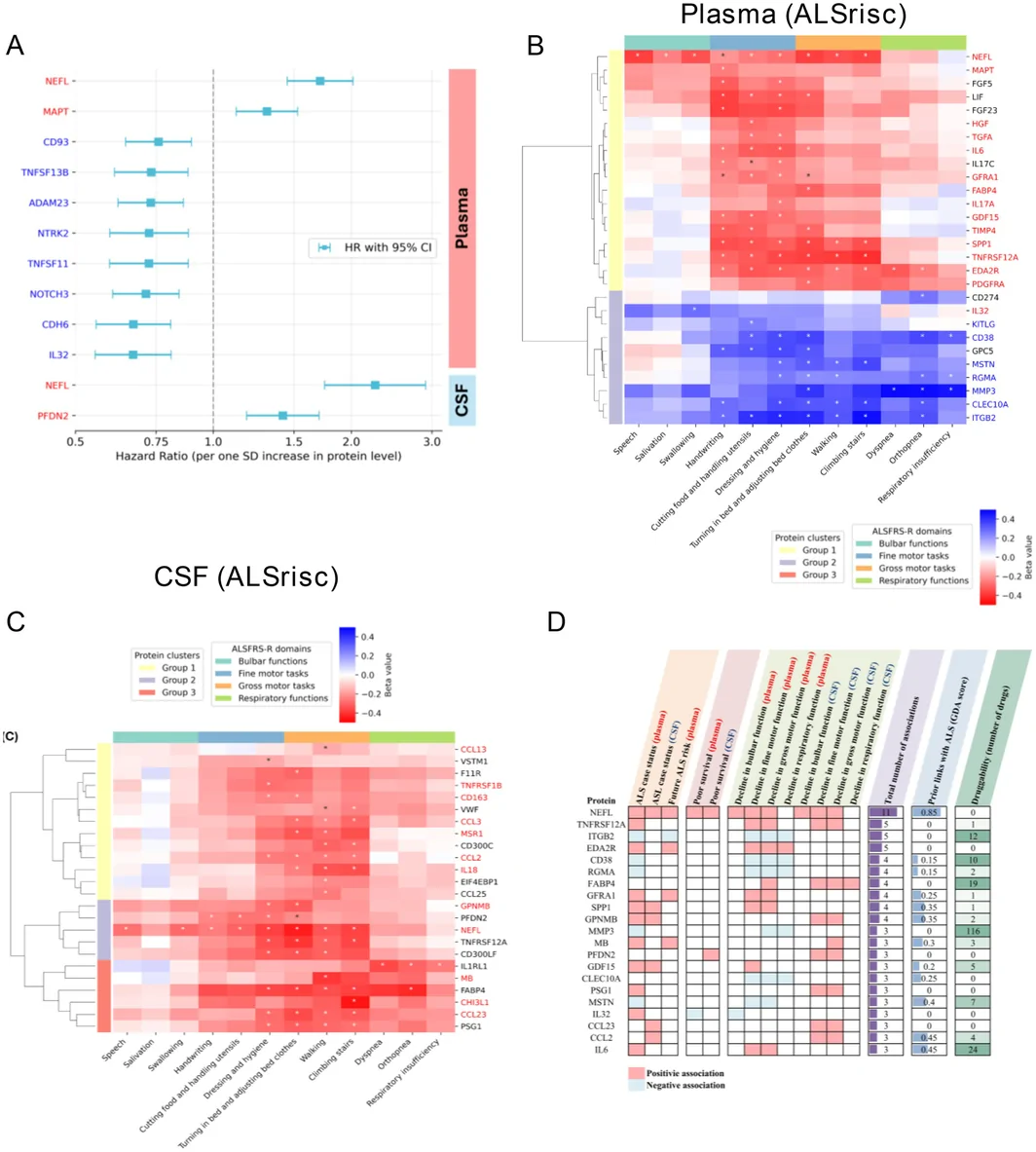
Proteins associated with ALS prognosis and integrative evidence summary (A): Forest plot demonstrating hazard ratios (HR) for mortality per 1 standard deviation (SD) increase in protein levels measured at diagnosis derived from a follow‐up analysis of ALS cases in the ALSrisc Study. Plasma proteins (top) and CSF proteins (bottom) with an FDR < 0.05 are shown with 95% confidence intervals. (B,C): Heatmaps of plasma proteins (B) and CSF proteins (C) whose levels measured at diagnosis predicted rate of decline in the 12 functional domains of ALSFRS‐R. Colors indicate effect sizes derived from mixed‐effects models (red = faster decline in relation to higher protein level; blue = faster decline in relation to lower protein level). Asteriss denotes an FDR < 0.05. Label colors of the Y‐axis indicate the significance and association direction of each protein with ALS case status (red = significant positive association, blue = significant negative association, black = no significant association). Hierarchical clustering partitioned proteins into different groups based on their domain‐specific association profiles. (D): Integrative evidence matrix for 21 proteins significant across three or more ALS outcomes. Columns 1–13 display positive (red) or negative (blue) associations for ALS case status, risk of future ALS, and survival and functional decline post‐diagnosis in plasma and CSF. Purple bars (column 14) indicate the total number of significant associations; column 15 shows DisGeNET gene‐disease association scores for ALS; column 16 lists the number of drugs targeting each protein according to the ChEMBL database.

We also identified proteins associated with ALS prognosis in ALSrisc. Ten plasma proteins were significantly associated with survival (FDR < 0.05; Table S8, Figure 3A). Higher levels of NEFL and MAPT were associated with a higher mortality risk, while the opposite was true for the remaining eight proteins. In CSF, NEFL and PFDN2 levels showed positive associations with mortality risk (FDR < 0.05; Table S9, Figure 3A).

To assess whether protein associations with rates of functional decline were domain‐specific, we examined domain‐specific rates of functional decline in ALS patients in 12 subdomains, which were further summarized into bulbar, fine/gross motor, and respiratory categories. At FDR < 0.05, in plasma, 26 proteins were associated with decline in fine or gross motor functions, including NEFL, MAPT, FGF5, CLEC10A, ITGB2, LIF, GPC5, EDA2R, SPP1, FGF23, IL6, IL17C, TNFRSF12A, GDF15, GFRA1, TIMP4, KITLG, HGF, TGFA, CD38, IL17A, MSTN, RGMA, MMP3, PDGFRA, and FABP4. Fewer plasma proteins were associated with bulbar decline (NEFL and IL32) or respiratory worsening (MMP3, EDA2R, CLEC10A, ITGB2, CD274, RGMA, and CD38) (Table S10, Figure 3B). In CSF, 23 proteins were associated with fine or gross motor decline, including NEFL, PFDN2, GPNMB, CCL23, TNFRSF12A, TNFRSF1B, CD300LF, CD163, PSG1, VSTM1, FABP4, CCL2, MSR1, F11R, IL18, CCL3, CCL13, VWF, EIF4EBP1, CD300C, CCL25, MB, and CHI3L1, whereas fewer were associated with bulbar decline (NEFL) or respiratory worsening (IL1RL1 and FABP4) (Table S11, Figure 3C).

In paired longitudinal samples, no plasma proteins showed significant within‐patient change after diagnosis (Table S12). In CSF, six proteins changed significantly over time (five increased, one decreased; Table S13). Notably, only PSG1 overlapped with the proteins previously linked to ALS disease status or functional decline.

### 3.4 Integrative Evidence Summary for ALS‐Related Proteins

To synthesize findings, we integrated association evidence for all 363 proteins examined in the ALSrisc Study with prior reports linking them to ALS and their potential druggability (Table S14). Among them, 125 (34.4%) were significantly associated with at least one ALS outcome in our analyses. Within this subset, 21 proteins provided strong and consistent evidence, each being significant across three or more distinct ALS outcomes, and the vast majority (except PSG1) remained longitudinally stable in plasma and CSF over the disease course (Figure 3D). Nine of the 21 proteins have not been previously linked to ALS in DisGeNET, and three of these nine proteins (PSG1, SPP1, and MMP3) have prior associations with frontotemporal dementia and/or dementia. Among these nine proteins, TNFRSF12A, ITGB2, FABP4, and MMP3 are predicted to be druggable targets in ChEMBL.

To address the potential mechanistic value of biofluid‐restricted associations, we summarized whether proteins associated with at least one ALS outcome showed evidence in plasma only, CSF only, or both biofluids. Among 125 proteins associated with at least one of these outcomes, 95 (76.0%) were plasma‐specific, 17 (13.6%) were CSF‐specific, and 13 (10.4%) showed evidence in both biofluids (Figure S10). The predominance of plasma‐specific proteins should be interpreted cautiously, as statistical power differed by biofluid in the ALSrisc Study. The highest‐evidence plasma‐specific proteins spanned a broader set of immune and extracellular matrix related processes, including leukocyte adhesion and activation markers (ITGB2, CD38), inflammatory signaling candidates (EDA2R, IL6), and extracellular matrix remodeling (MMP3). In contrast, the highest‐evidence CSF‐specific proteins were more tightly aligned with chemokine signaling and myeloid lineage biology, including the CC chemokines CCL2 and CCL23, and the monocyte or macrophage markers CD163 and CD300LF.

### 3.5 Bioinformatic Insights Into ALS‐Related Proteins

**FIGURE 4:**
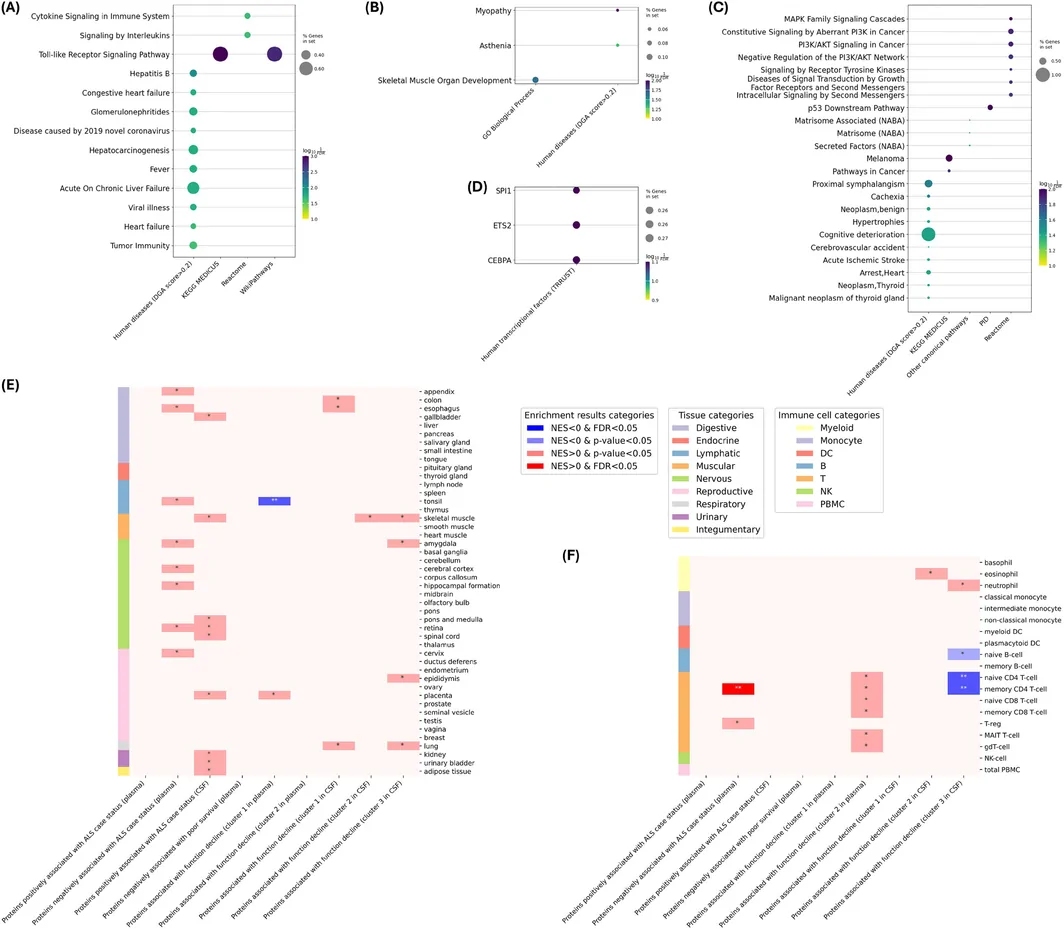
Enrichment analysis results for ALS‐related protein sets (A‐D): Bubble plot of significantly enriched canonical pathways, disease associations, and transcription factors for the 54 plasma proteins positively associated with ALS case status (A), ten plasma proteins positively associated with risk of future ALS (B), 18 plasma proteins in cluster 1 associated with functional decline (C), and 13 CSF proteins in cluster 1 associated with functional decline (D). Dot size indicates the percentage of input proteins in each gene set; color indicates −log10(FDR). (E‐F): Heatmaps of tissue‐specific expression enrichment (GSEA on 46 human tissues transcriptome) (E) and immune‐cell‐type specificity enrichment (GSEA on 18 immune cell transcriptomes) (F) across multiple ALS‐related protein sets (rows: Protein sets; columns: Tissues or immune‐cell types). Colors indicate normalized enrichment scores (NES), where red indicates positive and blue indicates negative NES, as well as significance level (p < 0.05, annotated with one asterisk, or FDR < 0.05, annotated with two asterisks).

PPI analysis in the ALS‐specific protein sets revealed two MCODE modules enriched in interleukin signaling pathways (Figure S11). Pathway enrichment analyses (Table S15) revealed that the ALS‐specific plasma proteins were strongly enriched for innate pathogen recognition, inflammatory signaling, and adaptive immune modulation pathways, as well as diseases characterized by inflammation, tissue injury, and metabolic stress (Figure 4A). Plasma proteins associated with higher future ALS risk were enriched in skeletal muscle developmental pathways and conditions characterized by muscle weakness and primary myopathies (Figure 4B). Plasma proteins associated with faster decline in motor functions were enriched in extracellular matrix organization and growth factor signaling pathways (Figure 4C). In addition, the CSF proteins associated with faster motor functions decline were enriched for targets of immune‐regulating TFs (Figure 4D).

Transcriptomic enrichment analysis showed that the genes encoding the plasma proteins linked to rapid motor decline were significantly depleted among genes with high RNA expression in palatine tonsil (Table S16, Figure 4E). In immune cell specific RNA profiles, genes encoding the plasma proteins negatively associated with ALS case status were enriched in T‐cell transcripts, whereas those encoding the CSF proteins linked to faster functional decline were depleted in T‐cell transcripts (Table S17, Figure 4F).

### 3.6 Plasma Protein Biomarkers Enhance ALS Risk Prediction

**FIGURE 5:**
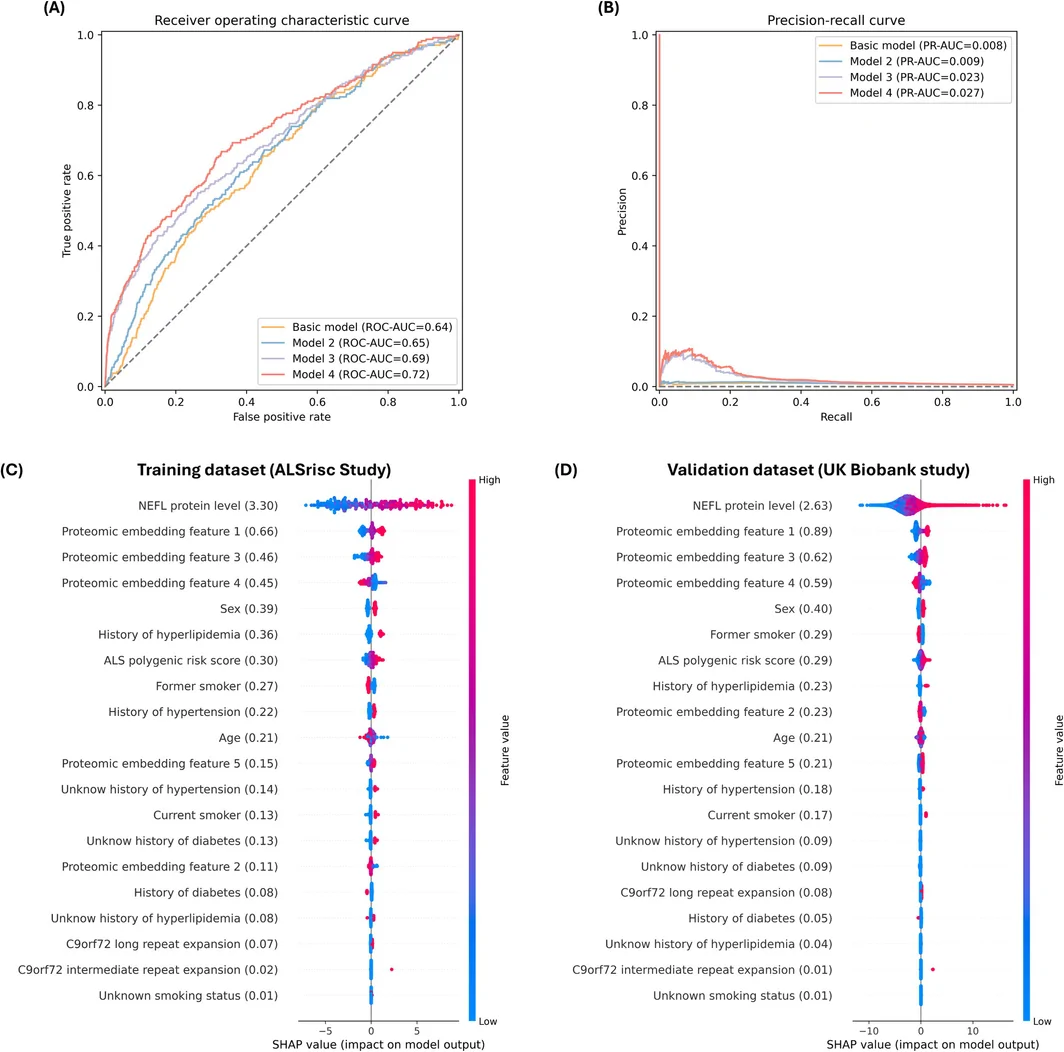
Performance of ALS risk prediction models and feature importance (A‐B): Receiver‐operating characteristic curves (A) and precision‐recall curves (B) of the four ridge logistic regression models for predicting risk of future ALS in non‐C9orf72 carriers in the UK Biobank study. The basic model included age, sex, BMI, smoking status, and medical history of hyperlipidemia, hypertension, and diabetes as predictors; Model 2 was based on Model 1 and further included ALS polygenic risk score and C9orf72 expansion group as predictors; Model 3 was based on Model 2 and further included plasma NEFL level; and Model 4 (full model) was based on Model 3 and further included five protein‐profile embeddings that represent levels of additional 356 plasma proteins. (C,D): SHAP summary plots for the full model (Model 4) applied to the ALSrisc Study training dataset (C) and the UK Biobank study validation dataset (D).

The basic model including only conventional risk factors achieved a validation ROC‐AUC of only 0.64 (PR‐AUC 0.008), which improved to 0.65 (PR‐AUC 0.009) with the addition of genetic predictors. Including NEFL boosted the ROC‐AUC to 0.69 (PR‐AUC 0.023), and the full model with five protein‐profile embeddings achieved ROC‐AUC of 0.72 (PR‐AUC 0.027, Figure 5A,B). Similar performance was observed when restricting ALS patients to those diagnosed at least 1 year after baseline in the validation cohort (ROC‐AUC of 0.71 and PR‐AUC of 0.021).

SHAP value analysis of the final model confirmed that NEFL was the single strongest predictor of ALS risk (Figure 5C,D). The next most influential features were three of the protein‐profile embedding variables and sex, with smoking and age showing modest contributions.

## 4 Discussion

In this large, multi‐cohort Olink protein profiling study, we identified plasma and CSF proteins associated with ALS case status, future risk, survival, and functional decline, and demonstrated that most candidate markers, including NEFL, are longitudinally stable over the disease course. We confirmed several established biomarkers, most notably NEFL, GPNMB, MSTN, SPP1, MB, and IL6, and highlighted a subset of 21 proteins with consistent associations across multiple ALS outcomes, including novel candidates such as TNFRSF12A, EDA2R, and FABP4. NEFL demonstrated the strongest and most consistent associations with ALS outcomes and emerged as the single most important predictor in the risk‐prediction model, reinforcing its central role as a core ALS biomarker.

Pathway, protein–protein interaction, and transcriptomic enrichment analyses suggested that ALS‐related protein signatures varied by biofluid and clinical outcome. Plasma proteins associated with ALS case status were mainly enriched for inflammatory and immune‐modulatory pathways, whereas plasma proteins associated with higher future ALS risk pointed toward skeletal muscle and myopathy‐related processes. For proteins associated with faster motor‐function decline, plasma findings highlighted extracellular matrix organization and growth factor signaling, while CSF findings implicated immune‐regulatory transcriptional programs. However, tissue and immune‐cell enrichment results were heterogeneous and should be interpreted cautiously. Rather than indicating a single shared ALS pathway profile, these analyses suggest that different protein sets may capture distinct aspects of ALS biology, including immune activation, muscle involvement, extracellular matrix remodeling, and CNS‐related immune regulation.

From a translational perspective, our results suggest that plasma protein measurements may contribute to future ALS risk stratification among non‐*C9orf72* carriers, although the incremental value beyond NEFL was modest. The ROC‐AUC of the model based on conventional risk factors was 0.64 and increased to 0.69 after the addition of NEFL. The full model incorporating five Olink protein‐profile embeddings further increased the ROC‐AUC to 0.72, indicating that a substantial proportion of the improvement was driven by NEFL, with a smaller additional gain from broader Olink‐derived protein features. Thus, the predictive value of the Olink protein‐profile embeddings should be viewed as complementary to, rather than independent of, the established NEFL signal. Integration with complementary omics approaches, such as transcriptomics, metabolomics, lipidomics, or single‐cell/spatial omics, may help provide a more comprehensive view of ALS biology by capturing molecular processes not fully represented by plasma protein profiles alone. In particular, single‐cell and spatial omics could help resolve the cellular sources and heterogeneity underlying bulk protein signals detected by Olink profiling.

Strengths of this study include the integration of a deeply phenotyped ALS case–control study with longitudinal plasma and CSF sampling and a large prospective population cohort with harmonized Olink protein‐profile and genetic data, enabling robust discovery and external validation across multiple ALS outcomes. The combination of clinical associations, network and enrichment analyses, and risk modeling provides complementary evidence for both mechanistic relevance and translational potential.

Limitations include the restricted protein coverage of the selected Olink panels in the ALSrisc Study, leaving many potential disease‐associated proteins unexplored. Although cross‐study comparisons with studies using Olink or SomaScan provide some reassurance regarding reproducibility, definitive analytical validation of individual proteins would require targeted immunoassays or mass spectrometry in independent samples. Olink PEA measurements are also susceptible to pre‐analytical variations, including residual platelet contamination, freeze–thaw cycles, and storage duration, although principal component analysis and pre‐analytical handling variation score analysis in the QC step did not suggest that such artifacts were a major source of variation in ALSrisc. Additional limitations include limited external replication of CSF and prognostic findings and the use of relative/family controls instead of population controls in ALSrisc. Moreover, the observational design of the study precludes causal inference. Experimental studies are needed to validate the functional roles of the implicated proteins and clarify whether they represent causal mediators, downstream markers, or both.

In conclusion, our study expands the catalog of ALS‐related proteins, identifies novel biomarker and therapeutic candidates, and supports ALS as a multi‐system disorder involving immune, muscle, and matrix remodeling pathways. The proof‐of‐concept risk‐prediction model demonstrates that plasma protein biomarkers can enhance ALS risk stratification years before diagnosis. Together, these findings highlight the value of incorporating protein biomarker assessment into ALS research and suggest avenues for earlier detection and disease monitoring.

## Author Contributions

**Lu Pan:** formal analysis, writing – original draft, writing – review and editing, visualization, investigation, validation, methodology, software. **Can Hou:** methodology, investigation, validation, formal analysis, visualization, writing – original draft, writing – review and editing, software. **Christina Seitz:** conceptualization, methodology, data curation, investigation, project administration, resources, writing – original draft, writing – review and editing, software. **Solmaz Yazdani:** writing – review and editing, investigation. **Caroline Ingre:** conceptualization, project administration, supervision, writing – review and editing, funding acquisition, investigation. **John Andersson:** investigation, writing – review and editing, data curation, project administration, resources, supervision. **Emily E. Joyce:** writing – review and editing, investigation. **Åsa K. Hedman:** investigation, writing – review and editing, formal analysis. **Charilaos Chourpiliadis:** investigation, writing – review and editing. **Jose Laffita:** writing – review and editing, investigation, methodology, formal analysis, data curation, resources. **Abbe Ullgren:** data curation, methodology, formal analysis, writing – review and editing, investigation, resources. **Sebastian A. Lewandowski:** investigation, writing – review and editing, funding acquisition, data curation, methodology, resources. **Trung Nghia Vu:** investigation, writing – review and editing, resources. **Yudi Pawitan:** investigation, writing – review and editing, conceptualization. **Anikó Lovik:** investigation, writing – review and editing. **Kristin Samuelsson:** conceptualization, investigation, writing – review and editing. **Myriam Barz:** validation, writing – review and editing, formal analysis, data curation, resources, project administration. **Fredrik Piehl:** investigation, conceptualization, methodology, supervision, writing – review and editing, resources. **Fang Fang:** conceptualization, investigation, funding acquisition, writing – original draft, writing – review and editing, visualization, validation, methodology, software, formal analysis, project administration, data curation, supervision, resources. **Rayomand Press:** writing – review and editing, resources, validation, investigation. **Oscar Fernandez‐Capetillo:** resources, data curation, formal analysis, writing – review and editing, validation. **Yan Chen:** investigation, writing – review and editing, formal analysis, resources. **Caroline Graff:** resources, supervision, funding acquisition, conceptualization, methodology, investigation, writing – review and editing. **Anders Mälarstig:** conceptualization, methodology, software, data curation, supervision, resources, project administration, formal analysis, visualization, validation, writing – review and editing, writing – original draft, funding acquisition, investigation.

## Funding

The study was supported by the H2020 European Research Council (MegaALS, 802091), the US Center for Disease Control and Prevention (R01TS000324 and R01TS000348), the Swedish Research Council (2023‐02428), Ulla‐Carin Lindqvist's Foundation, Bjorklunds fund, Neuro Sweden, and Konung Gustaf V:s och Drottning Victorias Frimurarestiftelse. In addition, contributions from SAL were supported by the Swedish Research Council (2021‐02605), Karolinska Institutet (2021‐01276), Fondation Thierry Latran (FIB‐ALS), Hjärnfonden (FO2022‐0233), Neuro (F2021‐0116), Bruno and Ilse Frick Foundation for Research on ALS, and Åhlen Stiftelse (223077).

## Conflicts of Interest

A.M. and A.K.H. were employees of Pfizer AB during the time of this work. Other authors declare no conflicts of interest.

## Supporting information


**Table S1:** Olink proteomic assays measured in the ALSrisc Study List of protein assays measured in the ALSrisc Study using four Olink Target 96 panels, i.e., Neuro Exploratory, Neurology, Cardiovascular III, and Inflammation. The table includes biosample type, Olink panel names, Olink assay ID, and protein assay name.
**Table S2:** Cross‐study Olink protein assay overlap across ALSrisc, UK Biobank, and Chia et al. List of Olink protein assays measured in ALSrisc and/or UK Biobank, with study‐specific panel/platform information and a flag indicating proteins in the provided ALSrisc–Chia et al. overlap list. ALSrisc used four Olink Target 96 panels; UK Biobank used Olink Explore.
**Table S3:** Quality control summary of ALSrisc Olink protein datasets *p*‐values for between group differences were calculated using analysis of variance or chi‐square tests, as appropriate.
**Table S4:** Clinical characteristics of ALS patients in the ALSrisc Study Baseline and follow‐up clinical data for 198 ALS cases in the ALSrisc Study.
**Table S5:** Plasma proteins associated with ALS case status in the ALSrisc Study GEE results for 363 plasma proteins (ALS cases vs. controls). Positive beta indicates higher levels in ALS cases.
**Table S6:** CSF proteins associated with ALS case status in the ALSrisc Study GEE results for 363 CSF proteins (ALS cases vs. controls). Positive beta indicates higher CSF levels in ALS cases.
**Table S7:** Plasma proteins associated with future ALS risk in the UK Biobank study Linear regression results for 2922 plasma proteins comparing baseline levels in individuals who later developed ALS vs. those who did not. Positive beta indicates higher baseline plasma levels in individuals who later developed ALS.
**Table S8:** Plasma proteins associated with survival in the ALSrisc Study Cox model results for 363 plasma proteins. Hazard ratio for all‐cause mortality is estimated as per 1 SD increase in plasma level.
**Table S9:** CSF proteins associated with survival in the ALSrisc Study Cox model results for 363 CSF proteins. Hazard ratio for all‐cause mortality is estimated as per 1 SD increase in CSF level.
**Table S10:** Associations between baseline plasma protein levels and functional decline by domain in the ALSrisc Study Mixed‐effects model results for 363 plasma proteins. Beta here indicates functional score change per 1 SD increase in plasma level, with negative beta indicating faster decline with increased plasma level. FDR < 0.05 is highlighted in red.
**Table S11:** Associations between baseline CSF protein levels and functional decline by domain in the ALSrisc Study Mixed‐effects model results for 363 CSF proteins. Beta here indicates functional score change per 1 SD increase in CSF level, with negative beta indicating faster decline with increased CSF level. FDR < 0.05 is highlighted in red.
**Table S12:** Longitudinal changes in plasma protein levels after ALS diagnosis in the ALSrisc Study Linear mixed‐effects results for plasma level change in 363 plasma proteins post‐diagnosis.
**Table S13:** Longitudinal changes in CSF protein levels after ALS diagnosis in the ALSrisc Study Linear mixed‐effects results for CSF level change in 363 plasma proteins post‐diagnosis.
**Table S14:** Integrated evidence matrix for ALS‐related proteins across outcomes For each of the 125 proteins listed with association with at least one ALS outcome, indicates direction of significant associations (*p*‐value < 0.05 for ALS risk or FDR < 0.05 for the rest outcomes) across 13 ALS outcomes (ALS status, risk, survival, functional decline).
**Table S15:** Pathway, disease‐association, and transcription factor targets enrichment analysis for ALS‐related protein clusters Enrichment results for predefined protein clusters against GO BP, KEGG, Reactome, DisGeNET, TF target databases. Significant level was set at FDR < 0.05 and > = 3 overlapping genes.
**Table S16:** Tissue‐specific gene expression enrichment for ALS‐related protein clusters GSEA results for each protein clusters against 46 human tissue transcriptomes. Significance level was set at FDR < 0.05.
**Table S17:** Immune cell‐type specificity enrichment for ALS‐related protein clusters GSEA results for each protein clusters against expression profiles of 18 immune cell types. Significance was set at FDR < 0.05.

**Figure S1:** Principal component analysis of plasma and CSF Olink NPX values by plate ID Scatter plots of the first two principal components (PCs) from PCA of z‐score standardized Olink NPX values, colored by assay plate. Each point represents one sample. (A) Plasma samples (PC1 16.0% variance, PC2 10.1% variance, plates 7 to 12). (B) CSF samples (PC1 29.3% variance, PC2 7.7% variance, plates 1 to 6). F‐test *p*‐values for associations between plate ID and PC1 or PC2 are shown in the panel headers.
**Figure S2:** Principal component analysis of plasma and CSF Olink NPX values by sample storage timeScatter plots of the first two principal components (PCs) from PCA of z‐score standardized Olink NPX values, colored by sample storage time (days). Each point represents one sample. (A) Plasma samples (PC1 16.0% variance, PC2 10.1% variance). (B) CSF samples (PC1 29.3% variance, PC2 7.7% variance). F‐test *p*‐values for associations between sample storage time and PC1 or PC2 are shown in the panel headers.
**Figure S3:** Principal component analysis of plasma and CSF Olink NPX values by ALS disease status Scatter plots of the first two principal components (PCs) from PCA of z‐score standardized Olink NPX values, colored by ALS case–control status (ALS, control). Each point represents one sample. (A) Plasma samples (PC1 16.0% variance, PC2 10.1% variance). (B) CSF samples (PC1 29.3% variance, PC2 7.7% variance). F‐test *p*‐values for associations between ALS disease status and PC1 or PC2 are shown in the panel headers.
**Figure S4:** Pre‐analytical handling variation scores for plasma and CSF by ALS disease status Violin plots of the pre‐analytical handling variation score, derived from proteins previously reported to be sensitive to pre‐analytical disturbance. Each point represents one sample; black dots and error bars indicate the mean and 95% confidence interval. (A) Plasma samples (ALS cases *n* = 176, controls *n* = 123, ANOVA *p*‐value = 0.09). (B) CSF samples (ALS cases *n* = 165, controls *n* = 14, ANOVA *p*‐value = 0.17).
**Figure S5:** Pre‐analytical handling variation scores for plasma and CSF by plate ID Violin plots of the pre‐analytical handling variation score, derived from proteins previously reported to be sensitive to pre‐analytical disturbance, stratified by assay plate. Each point represents one sample; black dots and error bars indicate the mean and 95% confidence interval. (A) Plasma samples (plates 7 to 12, ANOVA *p*‐value = 0.76). (B) CSF samples (plates 1 to 6, ANOVA *p*‐value = 0.54). Sample sizes for each plate are shown on the x‐axis.
**Figure S6:** Heatmaps of the 54 plasma proteins significantly increased in ALS in the ALSrisc study. Proteins were clustered by hierarchical clustering algorithm into five different groups (color bar at top) based on protein co‐expression patterns in ALS cases: Group 1 and 2: proteins primarily involved in neuroinflammatory processes; Group 3: proteins indicative of acute tissue injury; Group 4: proteins associated with coordinated repair and regenerative mechanisms; Group 5: exclusively represented by NEFL. Standardized protein NPX values are shown, with orange indicating higher and purple lower expression relative to mean values in controls.
**Figure S7:** Heatmaps of the 22 plasma proteins significantly decreased in ALS in the ALSrisc study. Proteins were clustered by hierarchical clustering algorithm into four different groups (color bar at top) based on protein co‐expression patterns in ALS cases: Group 1: negative regulators of muscle growth and neural guidance proteins; Group 2: extracellular matrix (ECM) components and cardiac/stress proteins; Group 3: TNF‐superfamily ligands and stem cell growth factors; Group 4: neural/epithelial adhesion molecules, proteases, and brain‐enriched matrix proteins. Standardized protein NPX values are shown, with orange indicating higher and purple lower expression relative to mean values in controls.
**Figure S8:** Cross‐platform concordance of ALS associated plasma protein signals between ALSrisc (Olink) and an independent SomaScan study. Scatter plot comparing per protein association statistics for ALS case–control differences in plasma between ALSrisc (Olink panel) and Dergai et al. (SomaScan panel). Each point represents one protein significant in ALSrisc at FDR < 0.05 and measured on both platforms (*n* = 71); red points indicate proteins increased in ALS cases in ALSrisc and blue points indicate proteins decreased in ALS cases. The solid line shows the fitted linear regression, with the shaded band indicating the 95% confidence interval. Proteins meeting FDR < 0.05 in both studies and showing the same direction of effect are labeled. Pearson correlation and *p*‐value are shown.
**Figure S9:** Heatmaps of the 10 CSF proteins significantly increased in ALS in the ALSrisc study. Proteins were clustered by hierarchical clustering algorithm into four different groups (color bar at top) based on protein co‐expression patterns in ALS cases: Group 1: nutrient sensing, protein clearance, and neurotrophic support proteins; Group 2: proteins of axonal damage with stress‐induced cytokines and chemokines; Group 3: proteins of microglia‐driven innate inflammation; Group 4: SPP1 only, involved in matrix remodeling and immunoregulation. Standardized protein NPX values are shown, with orange indicating higher and purple lower expression relative to mean values in controls.
**Figure S10:** Biofluid specificity of ALS associated proteins across outcomesVenn diagram summarizing proteins significantly associated with at least one ALS outcome (FDR < 0.05) in plasma only, CSF only, or both biofluids, based on outcomes available in both biofluids. Numbers indicate the number of proteins in each category. Protein labels are grouped by category, and label color denotes the evidence score (number of outcomes meeting FDR < 0.05).
**Figure S11:** Protein–protein interaction network of plasma proteins up‐regulated in ALS patients in the ALSrisc Study. A MCODE network analysis was applied to the 54 plasma proteins significantly elevated in ALS patients compared to controls (FDR < 0.05) in the ALSrisc Study. Two highly interconnected protein clusters were identified, both enriched for interleukin signaling pathways. Nodes represent proteins and edges indicate known interactions. Disconnected nodes in the network are not shown. Highlighted subnetworks reflect the significant clusters identified by the MCODE algorithm under default criteria.

**Data S1:** Supplementary Methods.

**Data S2:** Limit of detection (LOD) information for Olink assays. Assay‐specific LOD information for ALS plasma (panels A–D) and CSF samples (panels E–H) across the four Olink panels used in the ALSrisc study.

## Data Availability

Due to legal regulations, the data of the ALSrisc Study are not publicly available. Please contact the corresponding authors for more information. Data from the UK Biobank (http://www.ukbiobank.ac.uk/) are available to all researchers upon making an application.
