# Supplementary material for: Protein Biomarkers in Risk and Prognosis of Amyotrophic Lateral Sclerosis
Source: Eur J Neurol. 2026 Sep 5;33(9):e70741. doi: 10.1111/ene.70741 (PMC13545642; doi:10.1111/ene.70741)
Supplement: Supplementary file 1 [file ENE-33-e70741-s002.docx]

**Protein biomarkers in risk and prognosis of amyotrophic lateral sclerosis**

Lu Pan, Can Hou, Christina Seitz, Caroline Ingre, Åsa K. Hedman, Jose Laffita, Trung Nghia Vu, Yudi Pawitan, Abbe Ullgren, Solmaz Yazdani, John Andersson, Emily E. Joyce, Charilaos Chourpiliadis, Anikó Lovik, Yan Chen, Sebastian A. Lewandowski, Oscar Fernandez-Capetillo, Myriam Barz, Kristin Samuelsson, Rayomand Press, Fredrik Piehl, Caroline Graff, Anders Mälarstig, Fang Fang

**Supplementary Methods**

## **ALSrisc Study**

### ***Study design***

The ALSrisc (“Biomarkers and Risk Factors for ALS”) Study is an ongoing case-control study based in Stockholm, Sweden^1^. Since 2016, the study has enrolled newly diagnosed ALS patients from the Region of Stockholm, alongside disease-free full siblings and spouses of ALS patients as control participants. Patients were recruited at the ALS Clinical Research Centre of Karolinska University Hospital, where all received a diagnosis of probable, possible, or definite ALS according to the El Escorial criteria. ALS patients and their sibling or spouse controls were enrolled at the time of ALS diagnosis or shortly thereafter. The present analysis included 198 ALS patients, 78 sibling controls, and 47 spouse controls. The analysis of the ALSrisc Study was approved by the Swedish Ethical Review Authority (DNRs 2014/1815-31/4, 2018-1065/31, and 2021-06397-02). Informed consent, both oral and written, was obtained from all participants.

### ***Clinical information***

Using the unique personal identification numbers assigned to all residents in Sweden, we linked ALS patients to the Swedish Motor Neuron Disease (MND) Quality Registry. Since 2015, this registry has included information on most MND patients in Sweden, encompassing clinical characteristics, biological samples, and quality-of-life measures^2^. From the registry, we obtained data on date and age of diagnosis, sex, time and site of symptom onset, as well as the ALS Functional Rating Score-Revised (ALSFRS-R) and body mass index (BMI) measured at diagnosis and approximately every three months thereafter. For both cases and controls, we collected information on lifestyle factors (e.g., BMI and smoking), disease history (e.g., hypertension, diabetes, and hyperlipidemia), and medication use (e.g., antihypertensives), through a structured interview using a questionnaire modified from the “Euro-MOTOR” consortium^3^.

### ***Biological samples collection***

Blood and CSF samples were collected from ALS patients at diagnosis and in a subset of patients, annually thereafter. At each collection, 10 mL of blood (EDTA tube, BD) and 16 mL of CSF (two 10 mL plastic tubes, Sarstedt) were obtained. For controls, 10 mL of blood was collected at the time of recruitment, while a subset of controls recruited during the pilot phase in 2015 also provided a baseline CSF sample. Plasma was isolated by centrifuging blood samples at 2000 x g for 10 minutes at room temperature, while CSF samples were centrifuged at 400 x g for 10 minutes at 4^o^C. Samples were stored at -80^o^C till further analysis. DNA was extracted from 4 mL of frozen whole blood. Prior to conducting proteomic assays using the Olink Proteomics platform, both blood and CSF samples were thawed once to prepare 70 µL aliquots.

### ***Proteomic profiling***

Levels of 363 proteins from four Olink targeted panels (Olink Target 96 Neuro exploratory, Olink Target 96 Neurology, Olink Target 96 Cardiovascular III, Olink Target 96 Inflammation; **Supplementary Table 1**) were measured in plasma and CSF using the Olink proximity extension assay (PEA) (Olink Uppsala, Sweden)^4^ according to the standard protocols of the manufacturer for analyses and quality control^5^. Normalization was performed using the NPX Manager (Version 2.2.1.311), utilizing inter-plate controls to minimize technical variation. The resulting Normalized Protein eXpression (NPX) values, which represent relative normalized protein expression, were further standardized to z-scores based on the mean and standard deviation of the control participants. For both plasma and CSF, Olink quality-control (QC) flags were applied at the sample-protein measurement level. Individual protein measurements flagged as failing QC in specific samples were excluded or treated as missing for those samples. However, no protein assay was removed entirely at the protein level, i.e., all 363 Olink proteins were available across samples and retained for downstream analyses in both plasma and CSF. ALS patients withdrawn from the study were excluded. For samples with technical replicates, replicate NPX values were averaged protein-wise so that each individual contributed one value per biosample and time point. After sample-level quality control with technical replicates handling, the baseline case-control analyses included 176 ALS plasma samples and 123 control plasma samples, and 165 ALS CSF samples and 14 control CSF samples. Longitudinal measurements up to 36 months were available for 72 ALS patients in plasma and 39 in CSF.

### ***C9orf72 hexanucleotide repeat expansion***

We quantified the length of *C9orf72* G_4_C_2_ hexanucleotide repeat in both ALS patients and controls using a two-step protocol: fluorescent PCR followed by Triplet Repeat Primed PCR (AmplideX® PCR/CE C9orf72 Kit, Asuragen, Inc. Austin, TX)^6^. Individuals with ambiguous zygosity from the initial assay were re-analyzed with the Amplidex-C9. Based on repeat count of the larger allele, the participants were categorized into four expansion groups: short (≤6 repeats), long (7-24 repeats), intermediate (25-144 repeats), and expanded (≥145 repeats).

### ***Genotyping data***

Genome-wide genotyping was conducted for both ALS patients and controls using the Illumina Infinium Global Screening Array (GSAMD-24v3-0-EA_20034606_A1) on the SNP&SEQ Technology Platform in Uppsala, Sweden, yielding approximately 730,000 variants. We used the Sanger Imputation Service for standard pre-imputation quality control and imputation to approximately 23.8 million variants based on the 1000 Genomes Phase 3 reference panel. Post-imputation quality control involved the removal of variants with an information score<0.8, Hardy-Weinberg Equilibrium (HWE) p-value<1e-10, minor allele frequency (MAF)<0.01, or insertion-deletion variants. Additionally, participants of non-European ancestry were excluded. The final genetic dataset contained approximately 7.3 million high-quality single nucleotide polymorphisms (SNPs) across 290 participants (170 patients and 120 controls).

## **UK Biobank study**

### ***Study design***

The UK Biobank is a comprehensive cohort comprising approximately 500,000 middle-aged adults recruited across the UK^7^. Demographic and lifestyle information was collected at baseline through questionnaires and interviews, while health outcomes are continuously monitored via linkages to various national health registries in England, Wales, and Scotland, covering the period of 1997 to 2022. For the present analysis, we included 54,219 participants with available proteomic data following initial quality control. We excluded 23 individuals who had received an ALS diagnosis prior to blood collection, resulting a final cohort of 52,990 individuals. Among these, 298 participants were identified as incident ALS cases during follow-up based on registry data.

### ***Ascertainment of ALS***

The ascertainment of ALS in UK Biobank was conducted through inpatient hospital care and mortality records, using ICD-9 code 335.2 and ICD-10 code G12.2, which correspond to motor neuron disease (MND). Since ALS accounts for approximately 90% of all MND cases^8^, individuals with an MND diagnosis were considered as ALS cases for the purpose of this analysis.

### ***Proteomic profiling***

Proteomic profiling in the UK Biobank was conducted as part of the UK Biobank Pharma Proteomics Project (UKB-PPP) using the Olink Explore 3072 platform, which encompasses 2,923 unique plasma proteins (**Supplementary Table 1**). Details regarding sample handling and quality control for the proteomics data have been documented previously^9^. NPX values were obtained for 2,922 proteins that passed quality control (excluding GLIPR1). For this analysis, the NPX values of individual proteins were further standardized to z-scores based on the mean and standard deviation of the entire cohort.

### ***C9orf72 hexanucleotide repeat expansion***

We obtained size of *C9orf72* repeat expansion from the whole-genome sequencing short tandem repeat (STR) calls and we only used STR calls that passed quality control. Repeat length for the *C9orf72* locus (RapID “C9ORF72”) was extracted for all participants with available proteomics data, who were categorized into four expansion groups based on the repeat count of the larger allele, as described in the ALSrisc Study above.

### ***Genotyping data***

Participants in the UK Biobank were also genotyped using the UK Biobank Axiom Array, and the genotyping data were imputed to a reference panel that combines data from the Haplotype Reference Consortium, UK10K, and 1000 Genomes Project^7^. Imputed genetic data were extracted and subjected to post-imputation quality control, similar to the ALSrisc Study. We excluded individuals of non-European ancestry, those with sex mismatch or sex chromosome aneuploidies, those with insertion-deletion variants, and those with variants that had an info score<0.8, MAF<0.001, or HWE p-value<1e-10. The final genetic dataset for this analysis comprised approximately 9 million high-quality SNPs for 48,308 individuals.

## **Statistical analysis**

### ***Difference in protein levels between ALS patients and controls***

To identify proteins associated with ALS case status, protein levels at diagnosis (and time of recruitment for controls) were compared between ALS patients and controls in the ALSrisc Study. Plasma and CSF proteins were analyzed separately using a generalized estimating equation (GEE) model for each protein, accounting for within-family clustering among cases and controls. Adjustment was made for age, sex, technical factors (plate ID and sample storage time), and other covariates, including BMI, smoking, and medical history (hypertension, diabetes, and hyperlipidemia). In the UK Biobank, we employed linear regression to compare baseline plasma protein levels between incident ALS cases and other cohort participants, after adjusting for the same set of covariates. P-values were adjusted for multiple testing using the Benjamini-Hochberg (BH) False-Discovery Rate (FDR) method^10^, with an FDR<0.05 indicating statistical significance.

### ***Hierarchical clustering***

To group proteins with similar expression patterns among ALS cases, we performed agglomerative hierarchical clustering on normalized proteomic profiles from plasma and CSF in ALSrisc. Only proteins that were significant in the primary case control analyses were included. Linkage was computed using Ward linkage, and number of clusters were automatically determined using a maximum linkage distance criterion. Clustering was conducted separately for proteins with increased and decreased levels in ALS, and results were visualized with dendrograms and heatmaps to aid interpretation.

### ***Association between protein levels at diagnosis and disease prognosis***

In the ALSrisc Study, we followed ALS patients from the time of diagnosis until death or end of follow-up (January 31^st^, 2022). Initially, we used Cox proportional hazards models to estimate the hazard ratio (HR) of death per one standard deviation increase in the levels of various proteins measured at diagnosis, adjusting for age, sex, technical factors, BMI, smoking, disease history, and site of onset. We also assessed the relationship between protein levels at diagnosis and the rate of decline in ALSFRS-R scores post-diagnosis using a generalized additive mixed model. This model incorporated a cubic spline for time and a random intercept for each patient, adjusted for the same covariates. Plasma and CSF proteins were analyzed separately, with an FDR<0.05 used to indicate statistical significance.

### ***Longitudinal changes in protein levels after diagnosis***

Among the subset of patients with paired longitudinal proteomic data (72 for plasma and 39 for CSF proteins) in the ALSrisc Study, we examined the temporal trends in protein levels after diagnosis (up to 3 years) using a linear mixed-effects model. The model included a random intercept for each patient and was adjusted for age, sex, technical factors, BMI, smoking, disease history, and site of onset. A protein was deemed to have a statistically significant post-diagnosis temporal change if the time effect had an FDR<0.05.

### ***Evidence integration***

For proteins significantly associated with at least one ALS outcome, we queried the DisGeNET database^11^ to quantify their prior association strength with ALS, as indicated by the DisGeNET gene-disease association (GDA) score. Furthermore, we evaluated the druggability of these proteins by searching the ChEMBL database (release 35)^12^, classifying proteins as druggable if they were identified as known or predicted targets of approved drugs or those currently undergoing clinical trials.

### ***Bioinformatics analyses to characterize ALS-related proteins***

We conducted downstream bioinformatic analyses to characterize proteins significantly associated with various ALS outcomes, including ALS case status, risk, survival, and functional decline. First, we performed PPI network analysis using Metascape^13^, applying the Molecular Complex Detection (MCODE) algorithm to identify closely connected sub-networks (i.e., MCODE components). Each identified MCODE component was annotated with a representative functional description derived from ontology enrichment analysis. Next, we carried out functional enrichment analysis using GSEApy^14^ against gene sets from the Molecular Signatures Database^15^, TRUST^16^ and hTFtarget^17^ transcription factor databases, and the DisGeNET^11^ disease-gene association database. Genes corresponding to proteins assessed in the two respective studies were used as the background gene set. Significantly enriched terms were defined as those with an FDR<0.05 and at least three overlapping proteins. Additionally, we conducted gene set enrichment analyses (GSEA)^15^ to assess tissue specificity using transcriptomic profiles from 46 human tissues^18^ and 18 immune cell types^19^, with an FDR<0.05 for normalized enrichment score (NES) indicating statistical significance.

### ***Development and validation of ALS risk prediction model for non-C9orf72 carriers***

Finally, we developed a comprehensive ALS risk prediction model focusing on individuals without a *C9orf72* pathogenic expansion (<145 repeats). The model included a range of predictors, including demographic factors (age and sex), lifestyle factors (BMI and smoking), disease history (hypertension, diabetes, and hyperlipidemia), genetic factors (*C9orf72* repeat group within the non-expanded range [short, long, intermediate] and polygenic risk score [PRS] for ALS), and plasma protein levels (357 proteins measured in both studies). The ALS PRS was calculated based on summary statistics from the largest genome-wide association study (GWAS) of ALS in European ancestry population^20^ using the SBayesRC approach^21^. The *C9orf72* locus on chromosome 9 (coordinates 25,600,000-28,000,000) was excluded from this calculation. Among the proteins, NEFL was modelled as a separate predictor. To effectively handle missing data and reduce potential overfitting due to high dimensionality of data, the remaining 356 proteins underwent representation learning using a transformer-based foundation model, TabPFN^22^. This process generated a 192-dimensional latent feature vector, which was then reduced into five composite embeddings using Uniform Manifold Approximation and Projection (UMAP)^23^.

We constructed four prediction models incrementally: a basic model incorporating age, sex, BMI, smoking, and disease history; the basic model plus genetic factors; the basic model plus genetic factors and NEFL; and the basic model plus genetic risk factors, NEFL, and the five proteomic embeddings. Models were trained on the ALSrisc Study dataset using ridge logistic regression with a default L2 penalty and validated on the UK Biobank cohort. Model performance was evaluated using area under the receiver operating characteristic curve (ROC-AUC) and area under the precision-recall curve (PR-AUC). The contributions of predictors in the final model were assessed using the SHapley Additive exPlanations (SHAP) framework^24^. SHAP values were computed for each predictor in both the training (ALSrisc Study) and validation (UK Biobank study) datasets. For each feature, the mean absolute SHAP value across all individuals served as the importance score, with higher values indicating a stronger influence on the performance of the model.

## **References**

1. Chourpiliadis C, Seitz C, Lovik A, et al. Lifestyle and medical conditions in relation to ALS risk and progression-an introduction to the Swedish ALSrisc Study. *J Neurol* 2024;271(8):5447–59. doi: 10.1007/s00415-024-12496-w [published Online First: 2024/06/15]

2. Longinetti E, Regodón Wallin A, Samuelsson K, et al. The Swedish motor neuron disease quality registry. *Amyotroph Lateral Scler Frontotemporal Degener* 2018;19(7-8):528–37. doi: 10.1080/21678421.2018.1497065 [published Online First: 20181009]

3. D'Ovidio F, Rooney JPK, Visser AE, et al. Critical issues in ALS case-control studies: the case of the Euro-MOTOR study. *Amyotroph Lateral Scler Frontotemporal Degener* 2017;18(5-6):411–18. doi: 10.1080/21678421.2017.1285939 [published Online First: 20170210]

4. Assarsson E, Lundberg M, Holmquist G, et al. Homogenous 96-Plex PEA Immunoassay Exhibiting High Sensitivity, Specificity, and Excellent Scalability. *PLOS ONE* 2014;9(4):e95192. doi: 10.1371/journal.pone.0095192

5. Technology P. How is the Limit of Detection (LOD) estimated and how is this handled in the data analysis? : Olink; 2018 [Available from: <https://olink.com/faq/how-is-the-limit-of-detection-lod-estimated-and-handled/2023>.

6. DeJesus-Hernandez M, Mackenzie Ian R, Boeve Bradley F, et al. Expanded GGGGCC Hexanucleotide Repeat in Noncoding Region of <em>C9ORF72</em> Causes Chromosome 9p-Linked FTD and ALS. *Neuron* 2011;72(2):245–56. doi: 10.1016/j.neuron.2011.09.011

7. Sudlow C, Gallacher J, Allen N, et al. UK biobank: an open access resource for identifying the causes of a wide range of complex diseases of middle and old age. *PLoS Med* 2015;12(3):e1001779. doi: 10.1371/journal.pmed.1001779 [published Online First: 2015/04/01]

8. Rojas P, Ramirez AI, Fernandez-Albarral JA, et al. Amyotrophic Lateral Sclerosis: A Neurodegenerative Motor Neuron Disease With Ocular Involvement. *Front Neurosci* 2020;14:566858. doi: 10.3389/fnins.2020.566858 [published Online First: 2020/10/20]

9. Sun BB, Chiou J, Traylor M, et al. Plasma proteomic associations with genetics and health in the UK Biobank. *Nature* 2023;622(7982):329–38. doi: 10.1038/s41586-023-06592-6 [published Online First: 2023/10/05]

10. Benjamini Y, Hochberg Y. Controlling the False Discovery Rate: A Practical and Powerful Approach to Multiple Testing. *Journal of the Royal Statistical Society Series B (Methodological)* 1995;57(1):289–300.

11. Pinero J, Ramirez-Anguita JM, Sauch-Pitarch J, et al. The DisGeNET knowledge platform for disease genomics: 2019 update. *Nucleic Acids Res* 2020;48(D1):D845–D55. doi: 10.1093/nar/gkz1021 [published Online First: 2019/11/05]

12. Gaulton A, Hersey A, Nowotka M, et al. The ChEMBL database in 2017. *Nucleic Acids Res* 2017;45(D1):D945–D54. doi: 10.1093/nar/gkw1074 [published Online First: 20161128]

13. Zhou Y, Zhou B, Pache L, et al. Metascape provides a biologist-oriented resource for the analysis of systems-level datasets. *Nat Commun* 2019;10(1):1523. doi: 10.1038/s41467-019-09234-6 [published Online First: 2019/04/05]

14. Fang Z, Liu X, Peltz G. GSEApy: a comprehensive package for performing gene set enrichment analysis in Python. *Bioinformatics* 2023;39(1) doi: 10.1093/bioinformatics/btac757 [published Online First: 2022/11/26]

15. Subramanian A, Tamayo P, Mootha VK, et al. Gene set enrichment analysis: a knowledge-based approach for interpreting genome-wide expression profiles. *Proc Natl Acad Sci U S A* 2005;102(43):15545–50. doi: 10.1073/pnas.0506580102 [published Online First: 2005/10/04]

16. Han H, Cho JW, Lee S, et al. TRRUST v2: an expanded reference database of human and mouse transcriptional regulatory interactions. *Nucleic Acids Res* 2018;46(D1):D380–D86. doi: 10.1093/nar/gkx1013 [published Online First: 2017/11/01]

17. Zhang Q, Liu W, Zhang HM, et al. hTFtarget: A Comprehensive Database for Regulations of Human Transcription Factors and Their Targets. *Genomics Proteomics Bioinformatics* 2020;18(2):120–28. doi: 10.1016/j.gpb.2019.09.006 [published Online First: 2020/08/29]

18. Consortium F, the RP, Clst, et al. A promoter-level mammalian expression atlas. *Nature* 2014;507(7493):462–70. doi: 10.1038/nature13182 [published Online First: 2014/03/29]

19. Uhlen M, Karlsson MJ, Zhong W, et al. A genome-wide transcriptomic analysis of protein-coding genes in human blood cells. *Science* 2019;366(6472) doi: 10.1126/science.aax9198 [published Online First: 2019/12/21]

20. van Rheenen W, van der Spek RAA, Bakker MK, et al. Common and rare variant association analyses in amyotrophic lateral sclerosis identify 15 risk loci with distinct genetic architectures and neuron-specific biology. *Nat Genet* 2021;53(12):1636–48. doi: 10.1038/s41588-021-00973-1 [published Online First: 2021/12/08]

21. Zheng Z, Liu S, Sidorenko J, et al. Leveraging functional genomic annotations and genome coverage to improve polygenic prediction of complex traits within and between ancestries. *Nat Genet* 2024;56(5):767–77. doi: 10.1038/s41588-024-01704-y [published Online First: 2024/05/01]

22. Hollmann N, Muller S, Purucker L, et al. Accurate predictions on small data with a tabular foundation model. *Nature* 2025;637(8045):319–26. doi: 10.1038/s41586-024-08328-6 [published Online First: 2025/01/09]

23. McInnes L, Healy J, Melville J. Umap: Uniform manifold approximation and projection for dimension reduction. 2018. <https://arxiv.org/abs/1802.03426>.

24. Guyon I, Luxburg UV, Bengio S, et al., eds. A Unified Approach to Interpreting Model Predictions. Advances in Neural Information Processing Systems; 2017; Long Beach, CA, USA. Curran Associates, Inc.
