# Supplementary material for: Protein Biomarkers in Risk and Prognosis of Amyotrophic Lateral Sclerosis
Source: Eur J Neurol. 2026 Sep 5;33(9):e70741. doi: 10.1111/ene.70741 (PMC13545642; doi:10.1111/ene.70741)
Supplement: Supplementary file 2 [file ENE-33-e70741-s004.docx]

**Protein biomarkers in risk and prognosis of amyotrophic lateral sclerosis**

Lu Pan, Can Hou, Christina Seitz, Caroline Ingre, Åsa K. Hedman, Jose Laffita, Trung Nghia Vu, Yudi Pawitan, Abbe Ullgren, Solmaz Yazdani, John Andersson, Emily E. Joyce, Charilaos Chourpiliadis, Anikó Lovik, Yan Chen, Sebastian A. Lewandowski, Oscar Fernandez-Capetillo, Myriam Barz, Kristin Samuelsson, Rayomand Press, Fredrik Piehl, Caroline Graff, Anders Mälarstig, Fang Fang

**Supplementary Figures**

**Supplementary Figure 1** Principal component analysis of plasma and CSF Olink NPX values by plate ID

*Scatter plots of the first two principal components (PCs) from PCA of z-score standardized Olink NPX values, colored by assay plate. Each point represents one sample. (A) Plasma samples (PC1 16.0% variance, PC2 10.1% variance, plates 7 to 12). (B) CSF samples (PC1 29.3% variance, PC2 7.7% variance, plates 1 to 6). F-test p-values for associations between plate ID and PC1 or PC2 are shown in the panel headers.*

**Supplementary Figure 2** Principal component analysis of plasma and CSF Olink NPX values by sample storage time

*Scatter plots of the first two principal components (PCs) from PCA of z-score standardized Olink NPX values, colored by sample storage time (days). Each point represents one sample. (A) Plasma samples (PC1 16.0% variance, PC2 10.1% variance). (B) CSF samples (PC1 29.3% variance, PC2 7.7% variance). F-test p-values for associations between sample storage time and PC1 or PC2 are shown in the panel headers.*

**Supplementary Figure 3** Principal component analysis of plasma and CSF Olink NPX values by ALS disease status

*Scatter plots of the first two principal components (PCs) from PCA of z-score standardized Olink NPX values, colored by ALS case-control status (ALS, control). Each point represents one sample. (A) Plasma samples (PC1 16.0% variance, PC2 10.1% variance). (B) CSF samples (PC1 29.3% variance, PC2 7.7% variance). F-test p-values for associations between ALS disease status and PC1 or PC2 are shown in the panel headers.*

**Supplementary Figure 4** Pre-analytical handling variation scores for plasma and CSF by ALS disease status

*Violin plots of the pre-analytical handling variation score, derived from proteins previously reported to be sensitive to pre-analytical disturbance. Each point represents one sample; black dots and error bars indicate the mean and 95% confidence interval. (A) Plasma samples (ALS cases n = 176, controls n = 123, ANOVA p-value = 0.09). (B) CSF samples (ALS cases n = 165, controls n = 14, ANOVA p-value = 0.17).*

**Supplementary Figure 5** Pre-analytical handling variation scores for plasma and CSF by plate ID *Violin plots of the pre-analytical handling variation score, derived from proteins previously reported to be sensitive to pre-analytical disturbance, stratified by assay plate. Each point represents one sample; black dots and error bars indicate the mean and 95% confidence interval. (A) Plasma samples (plates 7 to 12, ANOVA p-value = 0.76). (B) CSF samples (plates 1 to 6, ANOVA p-value = 0.54). Sample sizes for each plate are shown on the x-axis.*

**Supplementary Figure 6** Heatmaps of the 54 plasma proteins significantly increased in ALS in the ALSrisc study.

*Proteins were clustered by hierarchical clustering algorithm into five different groups (color bar at top) based on protein co-expression patterns in ALS cases: group 1 and 2: proteins primarily involved in neuroinflammatory processes; group 3: proteins indicative of acute tissue injury; group 4: proteins associated with coordinated repair and regenerative mechanisms; group 5: exclusively represented by NEFL. Standardized protein NPX values are shown, with orange indicating higher and purple lower expression relative to mean values in controls.*

**Supplementary Figure 7** Heatmaps of the 22 plasma proteins significantly decreased in ALS in the ALSrisc study.

*Proteins were clustered by hierarchical clustering algorithm into four different groups (color bar at top) based on protein co-expression patterns in ALS cases: group 1: negative regulators of muscle growth and neural guidance proteins; group 2: extracellular matrix (ECM) components and cardiac/stress proteins; group 3: TNF-superfamily ligands and stem cell growth factors; group 4: neural/epithelial adhesion molecules, proteases, and brain-enriched matrix proteins. Standardized protein NPX values are shown, with orange indicating higher and purple lower expression relative to mean values in controls.*

**Supplementary Figure 8** Cross-platform concordance of ALS associated plasma protein signals between ALSrisc (Olink) and an independent SomaScan study.

*Scatter plot comparing per protein association statistics for ALS case-control differences in plasma between ALSrisc (Olink panel) and Dergai et al. (SomaScan panel). Each point represents one protein significant in ALSrisc at FDR < 0.05 and measured on both platforms (n = 71); red points indicate proteins increased in ALS cases in ALSrisc and blue points indicate proteins decreased in ALS cases. The solid line shows the fitted linear regression, with the shaded band indicating the 95% confidence interval. Proteins meeting FDR < 0.05 in both studies and showing the same direction of effect are labeled. Pearson correlation and p-value are shown.*

**Supplementary Figure 9** Heatmaps of the 10 CSF proteins significantly increased in ALS in the ALSrisc study.

*Proteins were clustered by hierarchical clustering algorithm into four different groups (color bar at top) based on protein co-expression patterns in ALS cases: group 1: nutrient sensing, protein clearance, and neurotrophic support proteins; group 2: proteins of axonal damage with stress-induced cytokines and chemokines; group 3: proteins of microglia-driven innate inflammation; group 4: SPP1 only, involved in matrix remodeling and immunoregulation. Standardized protein NPX values are shown, with orange indicating higher and purple lower expression relative to mean values in controls.*

**Supplementary Figure 10** Biofluid specificity of ALS associated proteins across outcomes

*Venn diagram summarizing proteins significantly associated with at least one ALS outcome (FDR < 0.05) in plasma only, CSF only, or both biofluids, based on outcomes available in both biofluids. Numbers indicate the number of proteins in each category. Protein labels are grouped by category, and label color denotes the evidence score (number of outcomes meeting FDR < 0.05).*

**Supplementary Figure 11** Protein-protein interaction network of plasma proteins up-regulated in ALS patients in the ALSrisc Study.

*A MCODE network analysis was applied to the 54 plasma proteins significantly elevated in ALS patients compared to controls (FDR<0.05) in the ALSrisc Study. Two highly interconnected protein clusters were identified, both enriched for interleukin signaling pathways. Nodes represent proteins and edges indicate known interactions. Disconnected nodes in the network are not shown. Highlighted subnetworks reflect the significant clusters identified by the MCODE algorithm under default criteria.*
